# The Patency of Tibial/Peroneal Arteries Affects the Increment of Regional Tissue Saturation of Oxygen in Each Angiosome after Superficial Femoral Artery Revascularization

**DOI:** 10.3400/avd.oa.21-00097

**Published:** 2022-03-25

**Authors:** Naoki Unno, Kazunori Inuzuka, Naoto Yamamoto, Masaki Sano, Kazuto Katahashi, Takafumi Kayama, Tatsuro Yata, Yuta Yamanaka, Hajime Tsuyuki, Yusuke Endo, Nozomu Ishikawa, Ena Naruse, Masatsugu Niwayama, Hiroya Takeuchi

**Affiliations:** 1 Division of Vascular Surgery, Hamamatsu University School of Medicine, Hamamatsu, Shizuoka, Japan; 2 Second Department of Surgery, Hamamatsu University School of Medicine, Hamamatsu, Shizuoka, Japan; 3 Division of Vascular Surgery, Hamamatsu Medical Center, Hamamatsu, Shizuoka, Japan; 4 Department of Electrical and Electronic Engineering, Shizuoka University, Hamamatsu Campus, Hamamatsu, Shizuoka, Japan

**Keywords:** ischemia, oxygen saturation, near-infrared spectroscopy, skin, TOE-20

## Abstract

**Objective**: The angiosome model is a controversial concept in the revascularization of patients with chronic limb-threatening ischemia (CLTI). The aim of this study was to demonstrate the importance of patency of the tibial/peroneal arteries for regional tissue oxygenation in each angiosome during endovascular therapy (EVT) of the superficial femoral artery (SFA).

**Materials and Methods**: We devised a novel near-infrared spectroscopy oximeter, “TOE-20,” for real-time monitoring of regional tissue oxygen saturation (rSO_2_). Using TOE-20, we prospectively assessed rSO_2_ at each angiosome in 23 CLTI patients who underwent successful revascularization of the SFA. During EVT, three sensor probes were placed at the dorsal foot, plantar foot, and outer ankle for rSO_2_ monitoring.

**Results**: At the end of EVT, rSO_2_ at all angiosomes was significantly elevated by SFA revascularization. The change in rSO_2_ in each angiosome was larger in patients with patent relevant arteries than in those with occluded relevant arteries (i.e., anterior tibial artery patency, posterior tibial artery patency, and peroneal artery patency).

**Conclusion**: The patency of the tibial/peroneal arteries is important for regional tissue oxygenation in EVT. Using TOE-20 and rSO_2_-based revascularization, it may possible to anticipate whether an ischemic ulcer will heal or not.

## Introduction

Growing incidence of diabetes and renal insufficiency has increased the number of patients with chronic limb-threatening ischemia (CLTI) to more than 6 million globally.^1)^ Catheter-directed angiography and endovascular therapy (EVT) are regarded as playing an important role among the treatment of CLTI, particularly in patients with a high surgical risk.^2)^

Angiosome is an each arterial perfusion area. Recently, it has been proposed for the improvement of tissue perfusion in critial limb ischemia patients’ feet using angiosome-guided EVT strategy.^3)^ This original concept was introduced by Taylor and Palmer in the planning of skin flaps based on the theory of arterial territories. The concept was investigated using fresh cadavers.^4)^ Both favorable and unfavorable results have been reported after angiosome-guided EVT.^5–7)^ Hence, the angiosome concept has not yet been established as a recommendable strategy for revascularization during EVT.

The discrepancy of the results after angiosome-guided EVT might be caused by the study design^2)^; most studies were performed in a retrospective manner with a limited number of patients. Furthermore, the outcomes were compared by the rate of wound healing or limb salvage between patients with direct (DR) and indirect (IR) revascularization, and evaluation of the results was performed 1 or 2 years after the procedure. None of the previous studies compared tissue perfusion in real-time between IR and DR during the EVT procedures, except one report, which measured skin perfusion pressure (SPP).^8)^ Traditional diagnostic modalities such as ankle-brachial index (ABI),^9)^ SPP,^10)^ and transcutaneous oxygen pressure^11,12)^ are difficult to use during EVT due to the limitations of the measurement site and long measurement time. The lack of proper diagnostic modalities to facilitate real-time monitoring of tissue perfusion prevents daily use of intra-EVT assessment.

Previously, we introduced a ﬁnger-mounted tissue oximeter using the near-infrared spectroscopy (NIRS) technique (Toccare; Astem Co., Ltd., Kawasaki, Japan) as a useful diagnostic device to assess the severity of ischemia in peripheral artery disease (PAD) patients.^13)^ In this study, we further develop an NIRS device to simultaneously facilitate the intra-EVT monitoring of tissue perfusion at multiple sites. Using the device, we report our initial results of tissue oxygenation at each angiosome during EVT.

## Materials and Methods

### Study approval

This study was approved by the Ethical Committee of the Hamamatsu University School of Medicine (approval number: 16-057) and Hamamatsu Medical Center (C014-2020). The study protocol was registered at the UMIN Clinical Trials Registry (UMIN-CTR; ID: UMIN000025021) and Japan Registry of Clinical Trials (CRB4180008). Written informed consent was obtained from all participants.

### TOE-20

**Figure 1:**
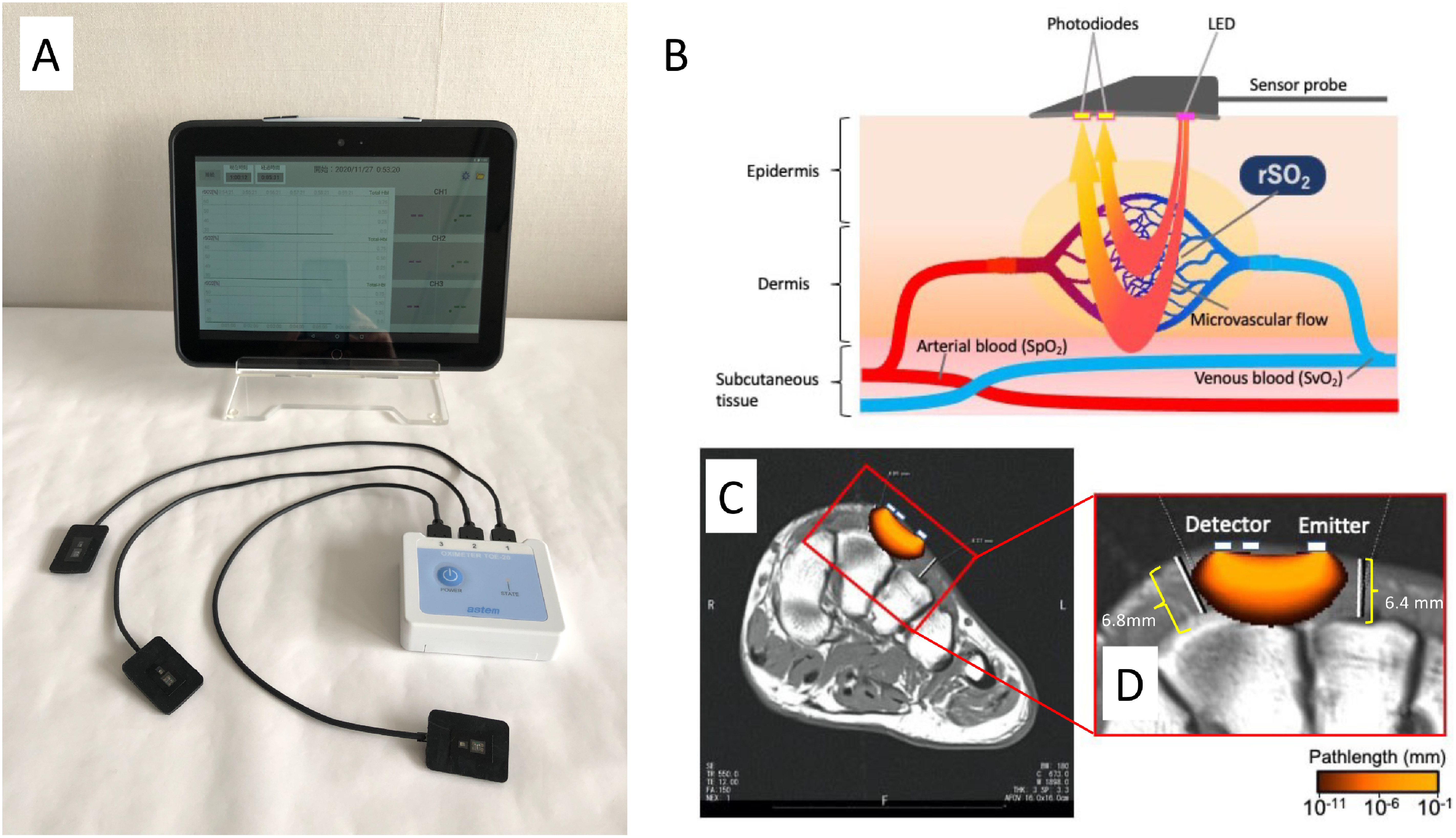
Fig. 1 (**A**) TOE-20. (**B**) Schematic diagram of the TOE-20 NIRS system to measure skin rSO_2_. (**C**) Path length distribution superimposed on a typical MRI image of foot. (**D**) Magnified image of the part surrounded by a red-colored square in **C**. The depth of bone from the skin surface is 6.4 to 6.8 mm at the dorsum of the foot.

MRI: magnetic resonance imaging; NIRS: near-infrared spectroscopy; rSO_2_: regional tissue oxygen saturation

Near-infrared light can penetrate body tissues to some depth. The new NIRS oximeter TOE-20 is manufactured by Astem Co., Ltd. for the purpose of real-time monitoring of tissue perfusion and has been recently commercialized (**Fig. 1A**). The oximeter has three sensor probes to simultaneously measure regional saturation of oxygen (rSO_2_) and can be connected to a tablet PC using Bluetooth. Each probe has near-infrared light emitting diodes (770 and 830 nm) and detectors (photodiodes). TOE-20 measures the amount of oxygenated and deoxygenated hemoglobin in the microvascular blood flow in both the epidermis and dermis tissues by determining light attenuation of the skin to which the sensor is attached, and calculates the rSO_2_ in approximately 0.5 s (**Fig. 1B**, Supplementary movie). **Figure 1C** shows the path length distribution of the near-infrared light superimposed on a typical MRI (magnetic resonance imaging) image of the dorsal foot, which was investigated by light propagation using a simulation model consisting of skin, fat, and bone layers.^14–17)^ As the optical path length shows, the measurement depth of TOE-20 is between 0 mm and 5 mm depth tissue from the skin surface.

### Theoretical analysis of NIRS

We investigated light propagation in a dorsal tissue model. The simulation model consists of skin, fat, and bone layers. The scattering coefficient μ_s_ and absorption coefficient μ_a_ were set to μ_s_skin_=26 mm^−1^, μ_s_fat_=24 mm^−1^, μ_s_bone_=40 mm^−1^, μ_a_skin_=0.02 mm^−1^, μ_a_fat_=0.003 mm^−1^, and μ_a_bone_=0.01 mm^−1^ based on literature data.^14–16)^ The anisotropic factors for each layer were 0.95.^17)^ The movement of photons in the Monte Carlo simulation was based on the radiative transport theory. The model was divided into 0.5×0.5×0.5 mm cubes, and the optical path lengths in each cube were determined by a weighted average using the light intensity when the photon reached the detector in order to examine measurement sensitivity. The optical path lengths were calculated when the source-detector distances were 6 and 8 mm, and it has been previously shown that the difference between the two optical path lengths is the measurement sensitivity in the spatially resolved method.^18)^ As the difference in optical path length in bone between the two states (source-detector distances: 6 or 8 mm) was minor and less than one-tenth of the total difference, it was shown that more than 90% of the total measurement sensitivity with the spatially resolved method would be related to hemodynamics in the skin and subcutaneous adipose tissues, at a depth of 0–5 mm.^19)^

In the measurement using TOE-20, the hemoglobin concentrations of both the skin and adipose tissues were measured. We built a lookup table of the relationship between the value of μ_a_ and the spatial slope of light intensity. In the measurement, the value of µ_a_ was calculated from the measured intensity slope using the lookup table. The following equations were used to calculate the concentrations of oxyhemoglobin ([O_2_Hb]) and deoxyhemoglobin ([HHb]): 
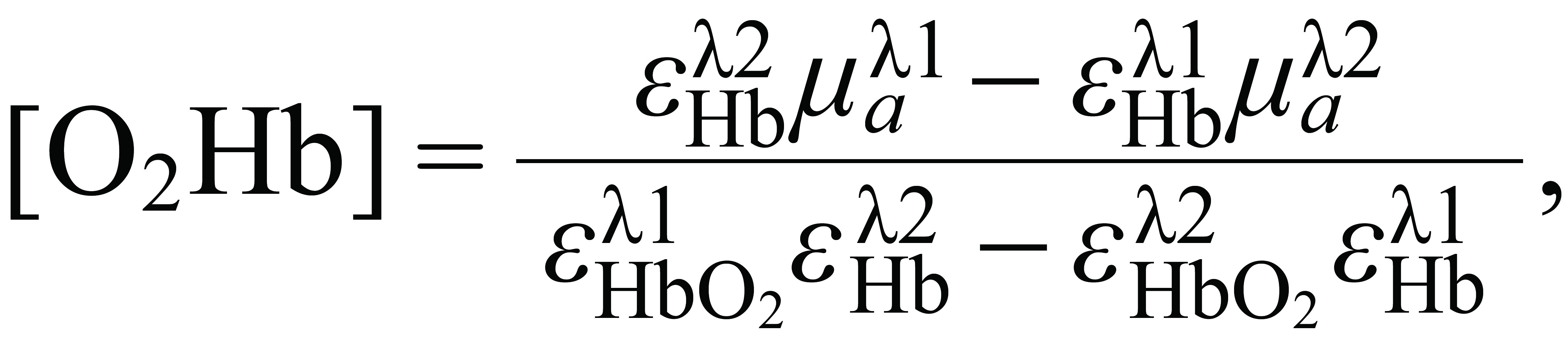
(1)
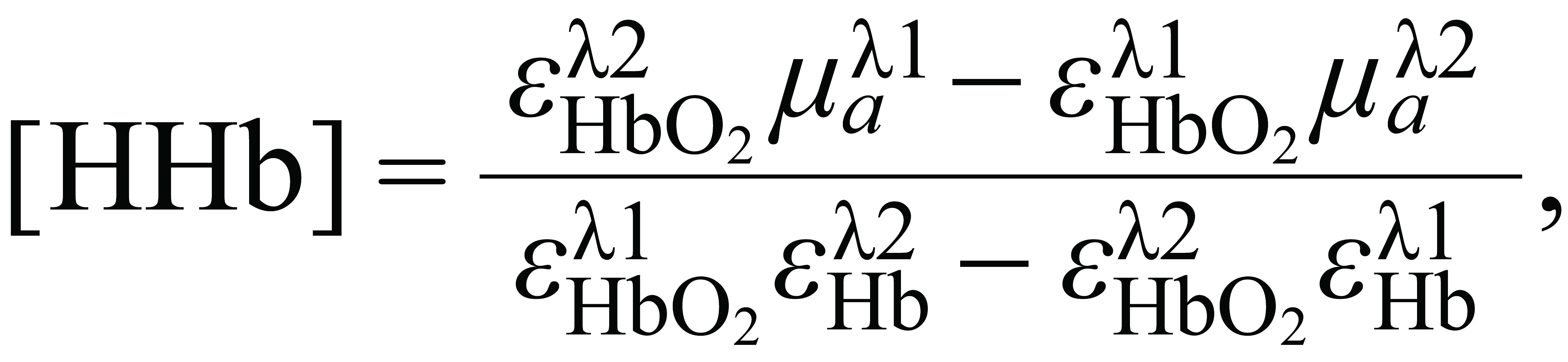
(2) where ε_HHb_^λ1,2^ and ε_O_2_Hb_^λ1,2^ are the extinction coefficients of HHb and O_2_Hb, respectively, at the wavelengths λ_1_ and λ_2_.^20)^ Regional tissue oxygen saturation rSO_2_ was calculated using the following equation:=[O_2_Hb]/([O_2_Hb]+[HHb]).

### Application of TOE-20 to patients

Three sensor probes can be placed at the operator’s discretion to monitor tissue perfusion. Sensor probes were placed according to the angiosome model as follows: the first one on the dorsal aspect of the foot, the second one on the outer ankle, and the last one on the plantar aspect of the foot.

### Subjects

This prospective study included 23 CLTI patients who underwent successful revascularization of the superficial femoral artery (SFA) without intervention of the anterior tibial artery (ATA), posterior tibial artery (PTA), and peroneal artery (PA) in two vascular centers between January 2020 and September 2020 (**Table 1**). All patients were categorized as Rutherford classification 5 with intractable foot ulcers and underwent measurement of the ABI, SPP (SensiLase PAD 3000; Vasamed, Inc., Eden Prairie, MN, USA), and rSO_2_ to evaluate the severity of the ischemia in the outpatient clinic. All patients also underwent pre-procedural imaging to confirm the diagnosis of chronic arterial occlusive disease (computed tomography arteriography and digital subtraction angiography). Successful revascularization of the SFA was defined as less than 30% residual stenosis of the target lesion on the completion angiogram without peripheral emboli.

**Table 1:** Table 1 Demographics and clinical characteristics of the patients

Patients, n	23
Age, years, median, interquartile range	78, 62–92
Men, n (%)	11 (48)
Hypertension, n (%)	13 (57)
Dyslipidemia, n (%)	10 (43)
Diabetes mellitus, n (%)	11 (48)
History of smoking, n (%)	15 (65)
End-stage renal disease, n (%)	15 (65)
Coronary artery disease, n (%)	5 (22)
Limbs, n	23
Location of ulcer	
Toe	20
Plantar foot	2
Outer ankle	1
Dorsal skin perfusion pressure, mmHg; mean±SD	31.3±13.7
Plantar skin perfusion pressure, mmHg, mean±SD	28.3±12.0
Dorsal foot tissue oxygen saturation, %, mean±SD	48.0±4.1
Plantar foot tissue oxygen saturation, %, mean±SD	49.3 ±2.9
Ankle-brachial index	0.57±0.33

SD: standard deviation

### Continuous rSO_2_ measurements during endovascular revascularization

Before the start of EVT, three sensor probes at the dorsal foot, plantar foot, and lateral ankle were placed on the patients’ skin surface. The rSO_2_ was measured in real-time from the beginning of the procedure to the end of EVT. rSO_2_ is the percentage of oxyhemoglobin [rSO_2_(%)=100×HbO_2_/(HbO_2_+Hb)]. rSO_2_ values were measured within 0.5 seconds at each region. Although rSO_2_ was monitored continuously, we waited 5 min to see the effect of the revascularization after each procedure because it took a few minutes for the value to stabilize. We recorded the rSO_2_ values of the three sensor probes placed at the dorsal foot, plantar foot, and the outer ankle at the end of EVT.

### Statistical analysis

Results are expressed as mean±standard deviation. Paired t-tests were used to compare rSO_2_ values between pre- and post-revascularization at the same sites. Wilcoxon signed-rank test was used to compare rSO_2_ values between the relevant artery-patent and artery-occluded groups. P-values <0.05 were considered statistically significant. Statistical analyses were performed with SPSS version 24.0 software (IBM Corp., Armonk, NY, USA).

## Results

### Patients’ demographics and rSO_2_ monitoring during EVT procedures

The baseline clinical characteristics of the study subjects are shown in **Table 1**. The numbers of ulcers were 20 at the toe, 2 at the plantar foot, and 1 at the outer ankle among the patients. SFA revascularizations were successfully performed by endovascular interventions with balloon angioplasty in 15 patients, Viabahn stentgraft (W. L. Gore & Associates, Flagstaff, AZ, USA) in 5, and stent placement in 3.

From the beginning to the end the EVT, monitoring of rSO_2_ at the dorsal foot, plantar foot, and the lateral ankle was successful in all patients. In most cases, rSO_2_ decreased mildly to be lower than that at the starting point during the intervention procedures, such as during balloon inflation and deflation. However, after SFA revascularization, the sensor probes gradually responded to the increased blood flow to the relevant area and increased rSO_2_ to a stable value.

### Interpretation of the completion angiography

**Figure 2:**
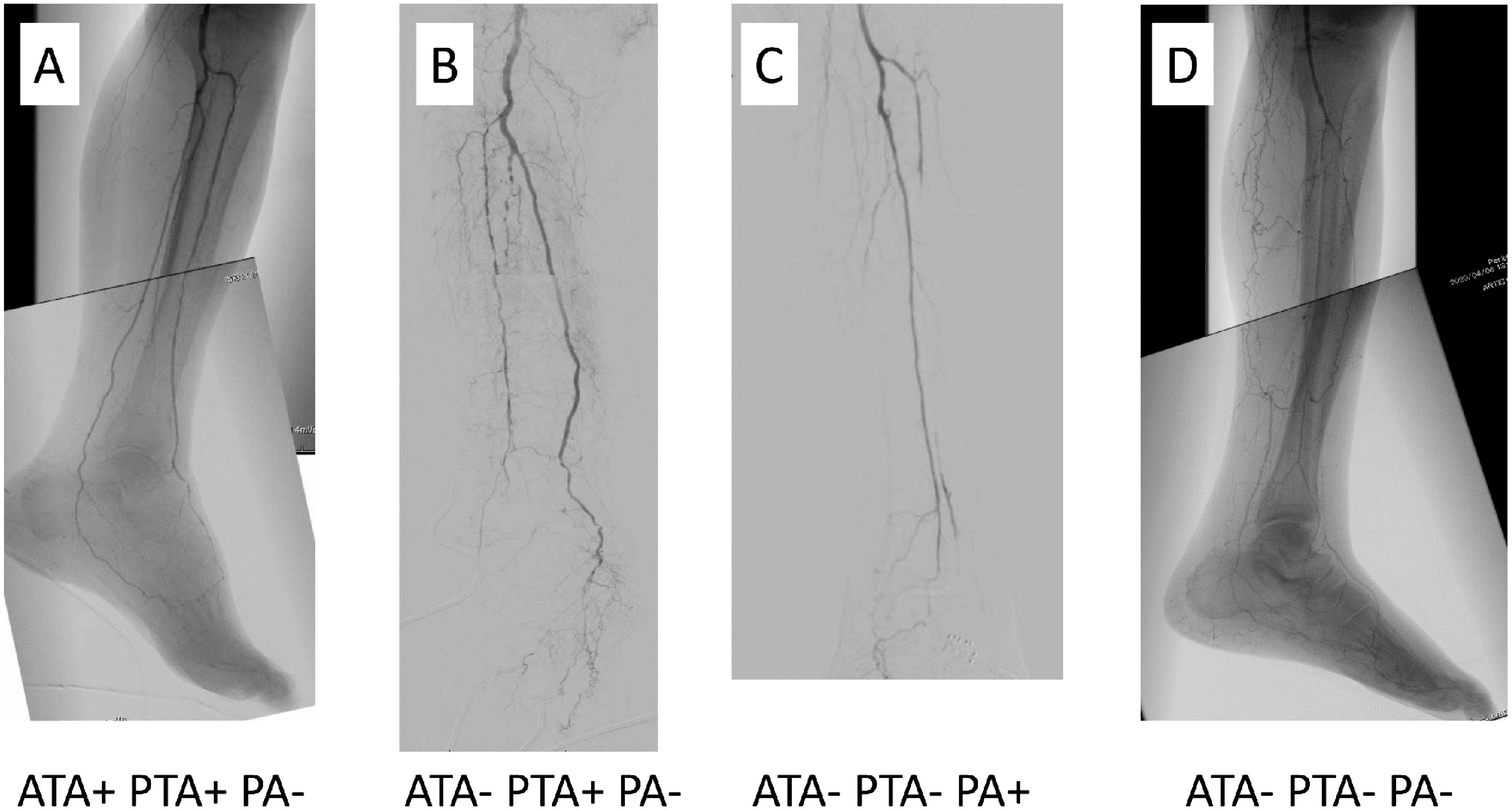
Fig. 2 Completion angiography. (**A**) Case where both ATA and PTA are patent (ATA+, PTA+), but the PA is occluded (PA−). (**B**) Case where both the ATA and PA are occluded (ATA−, PA−); only the PTA is patent (PTA+). (**C**) Case where both the ATA and PTA are occluded (ATA−, PTA−); only the PA is patent (PA+). (**D**) Case where all three arteries are occluded (ATA−, PTA−, PA−).

ATA: anterior tibial artery; PTA: posterior tibial artery; PA: peroneal artery

We interpreted the completion angiography after the final EVT procedures according to the patency of the ATA, PTA, and PA. **Figure 2** shows the typical patterns of arterial patency.

### rSO_2_ after SFA revascularization

**Figure 3:**
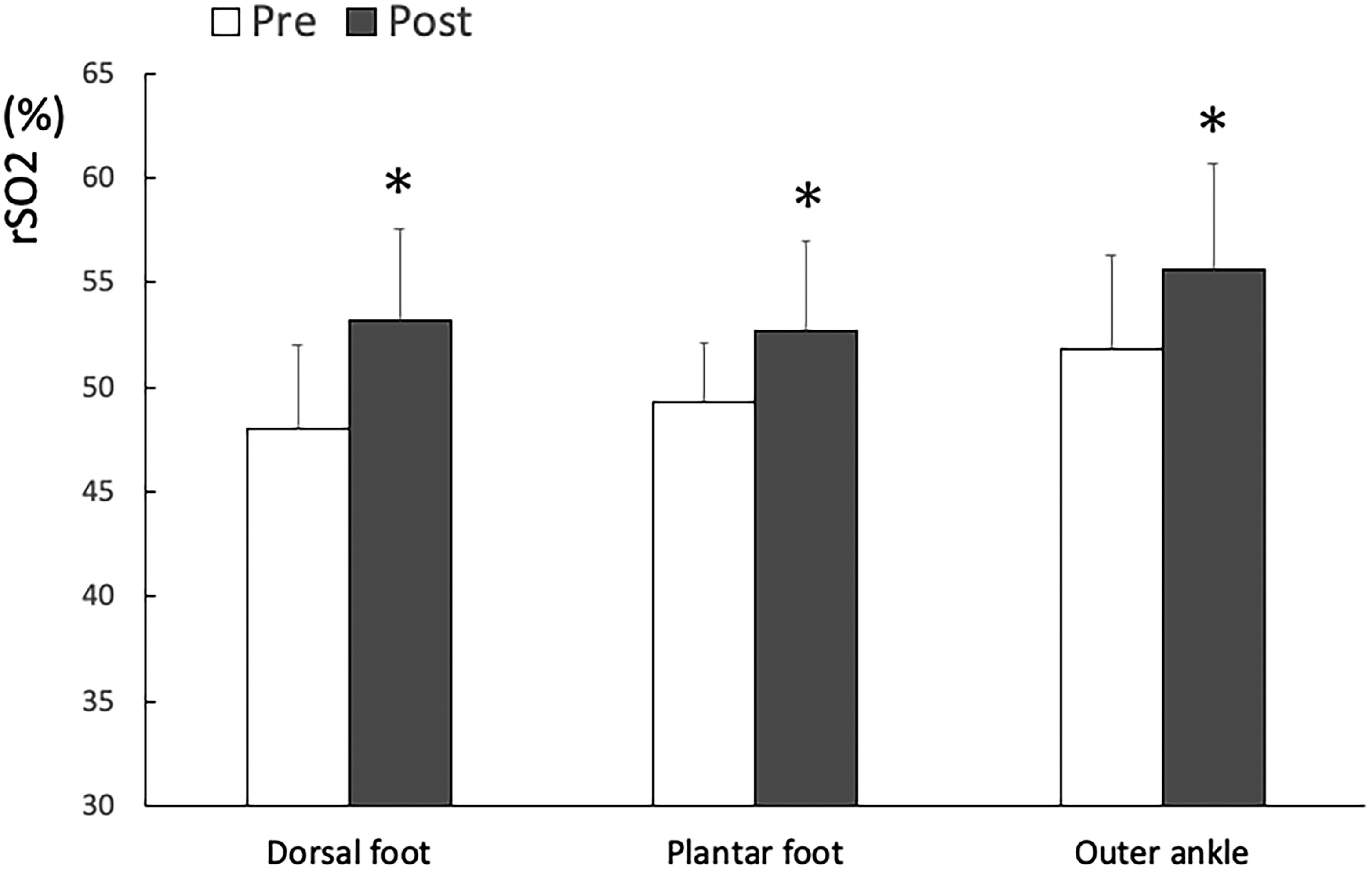
Fig. 3 rSO_2_ before and after SFA revascularization. * indicates p<0.01 in comparison with pre-EVT.

**Figure 4:**
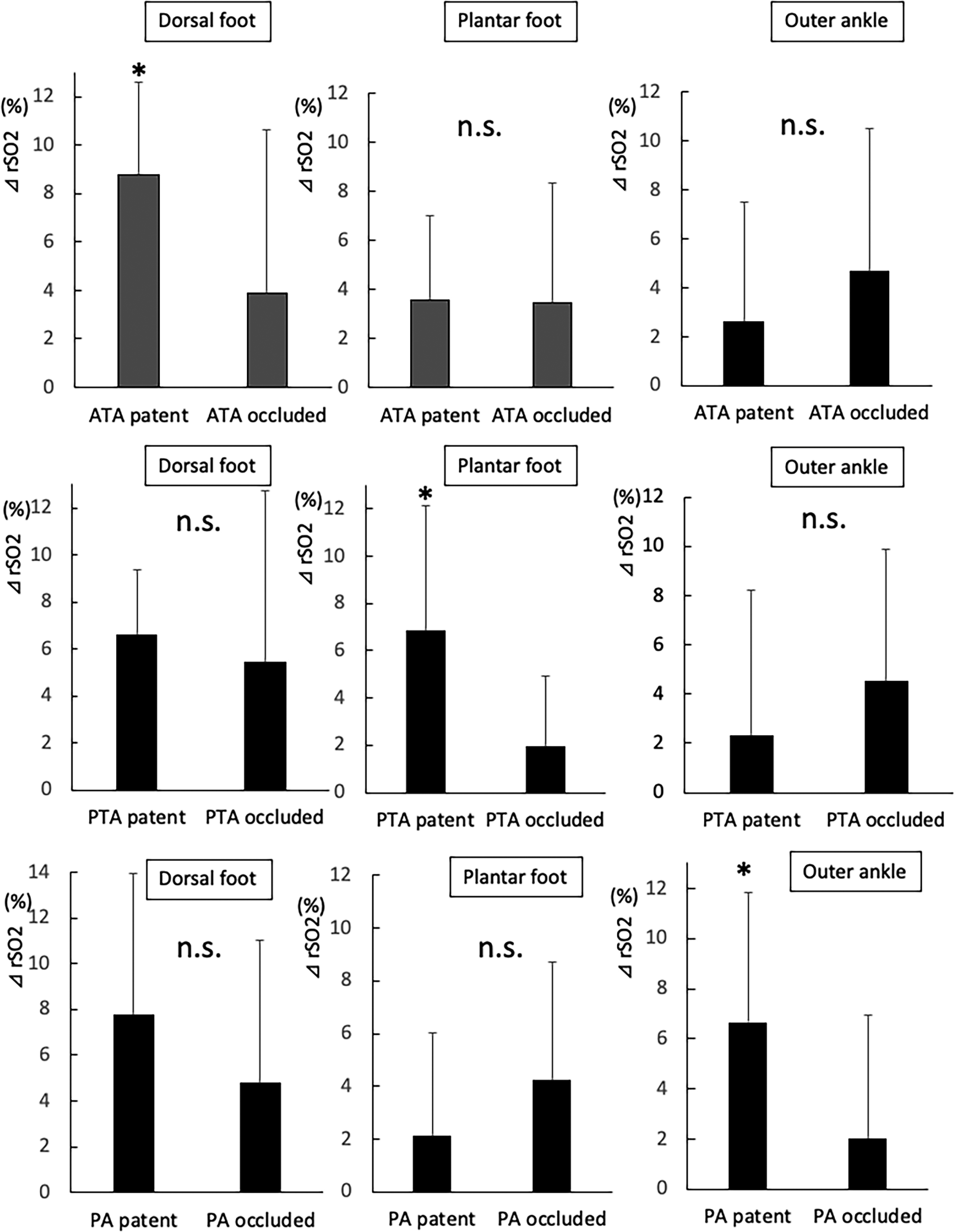
Fig. 4 Comparison of the amount of the increase (Δ) in rSO_2_ after SFA revascularization at the dorsal foot, plantar foot, and outer ankle between ΔrSO_2_ where the relevant arteries are patent or occluded. * indicates p<0.01 in comparison with pre-EVT; n.s., not significant.

ATA: anterior tibial artery; PTA: posterior tibial artery; PA: peroneal artery; rSO_2_: tissue oxygen saturation; SFA: superficial femoral artery; EVT: endovascular therapy

At the end of EVT, measurement of rSO_2_ with TOE-20 identified that all angiosome rSO_2_ values were significantly elevated by SFA revascularization in comparison with those at the beginning of EVT (48.0%±4.0% vs. 53.2%±5.6% at the dorsal foot, p<0.01; 49.3%±2.8% vs. 52.7%±4.3% at the plantar foot, p<0.01; 51.8%±4.5% vs. 55.6%±5.1% at the outer ankle, p<0.01) (**Fig. 3**). According to the angiosome model, the relevant artery in the dorsal foot is the ATA, that in the plantar foot is the PTA, and that in the outer ankle is the PA.^4)^ We further analyzed all values at the three areas, whether the relevant arteries were patent or not, at completion angiography by measuring the amount of the variation (Δ) in rSO_2_ at the end of EVT (**Fig. 4**). The amounts of ΔrSO_2_ were significantly larger when the relevant arteries were patent at all areas (i.e., dorsal foot and ATA+, plantar foot and PTA+, and outer ankle and PA+, compared to cases such as the dorsal foot and ATA−, plantar foot and PTA−, and outer ankle and PA−, respectively), while the ΔrSO_2_ was not significant even though the irrelevant arteries were patent.

rSO_2_: tissue oxygen saturation; SFA: superficial femoral artery; EVT: endovascular therapy

## Discussion

We have previously reported that our NIRS technique with a finger-mounted oximeter can diagnose the severity of PAD in a manner similar to that of traditional modalities such as ABI, SPP, and transcutaneous oxygen pressure.^13)^ We have also shown that TOE-20 demonstrated the utility to enable the monitoring of simultaneous perfusion in three angiosomes with three sensor probes during EVT.^21)^ The measurement depth of the previous NIRS oximeters was 10–20 mm under the skin surface to focus on brain or muscle oxygen levels^22)^; however, the skin/subcutaneous tissue of the toe might be too thin to measure because the bones or tendons are present at depths less than 10 mm from the skin surface. On the other hand, TOE-20 is specifically designed to measure tissue oxygen saturation focusing only on the skin and subcutaneous tissue. For that purpose, a simulation model consisting of skin, fat, and bone layers was created for algorithm development. The superimposed foot MRI image revealed that the rSO_2_ of TOE-20 reflects tissue oxygen levels up to 5 mm under the skin surface and measures rSO_2_ of the skin and subcutaneous tissue without the influence of bone. This characteristic is quite unique compared to other NIRS devices, which mainly measure cerebral oxygen levels using an algorithm that diminishes the contribution of the skin and scalp.^23)^ Because high and low rSO_2_ areas are seen even in the same angiosome, the peripheral tissue perfusion in the CLTI patient’s foot, especially diabetic patients, is determined by the peripheral microvascular blood flow in the skin and subcutaneous tissue.^24)^ Therefore, the use of TOE-20 may be applicable not only for foot tissue monitoring during EVT but also for assessment of free skin graft perfusion during plastic surgery.

Using TOE-20, we successfully monitored the changes of rSO_2_ at each angiosome simultaneously during EVT. The results showed that successful SFA revascularization significantly increased the oxygen levels at all angiosome areas irrespective of tibial artery patency. However, the amount of ΔrSO_2_ in each angiosome depended on relevant tibial artery patency. It is believed that the development of collateral blood flow is individually different in PAD patients, so that the angiosome model may not necessarily be applied to PAD patients with diseased tibial and peroneal arteries. Indeed, Kawarada et al. reported no differences between DR and IR of the relevant ATA or PTA in the amount of ΔSPP in the area of the dorsal and plantar foot after revascularization.^8)^ In this report, however, the results of the amount of ΔrSO_2_ after SFA revascularization showed that ΔrSO_2_ was significantly larger in angiosomes with patent relevant tibial/peroneal arteries than in those with occluded arteries, suggesting that even in patients with diseased tibial/peroneal arteries, the conventional angiosome model works. Therefore, when we perform revascularization of the SFA, improved oxygenation in the region where the relevant artery is patent can be anticipated. Moreover, in combination with tibial/PA interventions, the angiosome concept may be associated with improved perfusion at the region where ulcers are located.

While the angiosome-targeted EVT is planned by reference to pre/intra-EVT angiography, the completion angiogram at the end of EVT reflects increased perfusion because the intra-EVT angiogram may be affected by catheter/sheath insertion, causing perturbed blood flow. Because of this, we and others previously reported the usefulness of indocyanine green (ICG) fluorescence angiography at completion angiography for the assessment of technical success of the below-the-knee revascularization in either bypass surgery or EVT.^25–27)^ ICG fluorescence angiography allows visualization of the perfusion area with fluorescence signals obtained 5–8 mm deep to the skin surface, so that the effect of revascularization can be assessed in real-time; however, ICG fluorescence angiography has several limitations. First, the ICG injection cannot be repeated during EVT procedures because the fluorescence signals remain for a while due to ICG binding to the tissue proteins. Second, the cut-off values of the ischemic region for wound healing remain to be clarified. Third, patient cardiac function may affect the fluorescence signals. Several additional limitations such as the difference of infrared cameras, patient physique, and the area of the foot prevent the technique from being the standard modality for real-time assessment of perfusion. On the other hand, real-time monitoring of rSO_2_ using TOE-20 can overcome such problems. TOE-20 can repeatedly measure rSO_2_ at the site of probe placement after each EVT procedure. A previous study identified that an rSO_2_ of more than 50% may be a cut-off value for wound healing.^28)^ Patient cardiac function, physique, and skin color do not influence the rSO_2_ value. Therefore, TOE-20 may become an ideal diagnostic device to monitor tissue perfusion/oxygenation in real-time.

The device’s name, TOE, is an abbreviation for target region oxygenation-based endovascular treatment, which has previously been proposed as a new EVT strategy.^29)^ In TOE, we aim to adequately perfuse the target region up to rSO_2_>50%. For that purpose, the multi-channel oximeter, TOE-20, was designed to monitor tissue oxygenation because CLTI patients often possess multiple ulcers in different angiosomes. Using this method, we may be able to anticipate whether the ischemic ulcer will heal or not.

## Study Limitations

First, the number of patients who were investigated for the utility of TOE-20 was limited. Second, we did not show the follow-up data of the rate of patient wound healing as well as limb salvage in relation to the rSO_2_ values after EVT. Third, we did not include cases with tibial/PA revascularization but only those with SFA revascularization to observe changes in the ΔrSO_2_ values. Finally, some of the authors hold a patent associated with TOE-20, which might cause potential bias during result evaluation. Considering the abovementioned limitations, this study is still preliminary and further studies are needed with longer follow-up periods.

## Conclusion

We devised a novel oximeter, TOE-20, for real-time monitoring of tissue oxygenation during EVT for CLTI patients. TOE-20 demonstrated usefulness in the measurement of rSO_2_ values and identified the increase of perfusion after SFA revascularization in all angiosomes of the foot, irrespective of relevant tibial/PA patency. The amount of ΔrSO_2_ was significantly larger in the angiosomes with patent relevant tibial/peroneal arteries than in those with occluded arteries, which suggests that the angiosome model may be important, even in CLTI patients with diseased infra-popliteal arteries.

## Supplementary Information

Supplementary movie is available at the online article’s sites on J-STAGE and PMC.
